# Case of Rupture of Urethra

**Published:** 1863-02

**Authors:** S. Barrett

**Affiliations:** Le Roy


### ART. II.—*Case of Rupture of Urethra.*

[For the Buffalo Medical and Surgical Journal.]

I send you the following case of rupture of the membraneous portion of the urethra. I. U. D., aged 56, farmer, of Pavilion, fell while adjusting a girt in his corn barn, about eight feet, astride an inch board, bringing the membraneous portion of the urethra between the square edge of the board and the arch of the pubis. In about an hour after, he felt a desire to urinate; on making the effort, the urine all passed into the cellular tissue of the scrotum and perineum, which soon became filled with extravasated blood and urine, His family physician, Dr. U. Fay was sent for; who on arriving soon discovered the nature of the injury. A messenger was dispatched for me; I arrived at his house about twelve o'clock at night, some five hours after the injury, found him with his scrotum enormously distended, making great effort to void urine every five miuutes; I had him placed upon a table in the position for the operation for lithotomy; after bringing him fully under the influence of chloroform, I passed a catheter into the urethra down to the seat of injury, there made an incision about the same extent as for the lateral operation for lathotomy—laying the parts freely open; on com ing to the membraneous portion of the urethra the catheter passed freely out showing that portion of it to be entirely severed to the extent of nearly an inch; after removing the coagula, which had formed there so as to leave a free exit to the urine, we had him put to bed with the scrotum elevated to favor the escape of the extravasated urine and blood. We did not introduce a catheter immediately as is commonly directed in such cases, as the contused and lacerated parts would require considerable time to slough off, before the reparative process could commence. He passed his urine freely through the incision, without pain, for some six weeks. When the parts had taken a healthy action, and the space had begun to fill up, I passed a No. 8 silver catheter through the penis into the bladder and retained it there, by tapes and a bandage. The urine all passed through the catheter; the reparative process went on well, the granulation gradually closing up around the catheter, occasionally requiring a slight penciling with the nit. argent The catheter required to be removed occasionally and cleansed, as it became slightly encrusted with phosphate of lime. In about a year the parts had become nearly sound, and he was enabled to dispense with the catheter; by using occasionally an elastic bougie, we succeded in maintaining the urethra nearly of its normal size—and now being three years since the injury, he passes his urine freely, without being obliged to resort to any aid, and is in excellent health. The successful result of this case, I think, is very much to be attributed to not retaining the catheter in the bladder until the parts had taken on healthy action and began to granulate, and then of having one of proper size, that the new parts forming around it could not contract so as to leave the canal too small,

Yours Respectfully, S. BARRETT, M. D.

*Le Roy, Dec.* 30, 1862.

